# Associations between dietary patterns and blood pressure in a sample of Australian adults

**DOI:** 10.1186/s12937-019-0519-2

**Published:** 2020-01-14

**Authors:** Claire Margerison, Lynnette J. Riddell, Sarah A. McNaughton, Caryl A. Nowson

**Affiliations:** Deakin University Institute for Physical Activity and Nutrition, Locked Bag 20000, Waurn 11 Ponds, Geelong, VIC 3220 Australia

**Keywords:** Dietary patterns, Factor analysis, Blood pressure, Sodium, Potassium, 24-h recall

## Abstract

**Background:**

Investigating effects of whole diets on blood pressure (BP) can contribute to development of diet-based recommendations for health. Our aim was to assess the relationship between dietary patterns and BP in a sample of free-living Australian adults.

**Methods:**

Usual dietary patterns of participants recruited to dietary intervention studies were assessed using factor analysis (two 24-h recalls). The mean of seven days of daily, seated BP measurements were used.

**Results:**

Complete data from 251 participants (112 males; mean age 55.1(9.1) (SD) years; body mass index (BMI) 29.5(3.9) kg/m^2^) was included. Three dietary patterns were identified. Only Dietary Pattern 2 was positively associated with home systolic BP (β = 1.88, 95% CI 0.16, 3.60) after adjusting for age, sex, BMI, anti-hypertensive medication, smoking, education, physical activity and energy intake. This dietary pattern was characterised by high consumption of low-fibre bread, pasta, noodles and rice, meat dishes, poultry dishes and egg dishes, mixed cereal dishes, salted nuts and low consumption of milk and yoghurt (low-fat), vegetable juice, vegetables and high-fibre bread. Dietary Pattern 2 was also positively associated with intakes of energy (*P* = 0.002) and sodium (*P* = 0.005) and inversely associated with potassium intake (*P* = 0.002). After adjustment for energy, only the inverse association with potassium remained (*P* <  0.001).

**Conclusions:**

In this sample of Australian adults, Dietary Pattern 2 was associated with higher BP and thus chronic disease risk, supporting the evidence that diets high in energy and sodium, and low in potassium from vegetables and dairy, are detrimental to cardiovascular health.

## Background

Hypertension is the main risk factor in the development of cardiovascular disease (CVD) [1], and CVD is the number one cause of death globally [2]. The relationship between dietary intake and hypertension is complex. Individual nutrients involved in the development of hypertension have been extensively studied, and it is widely accepted that a decrease in sodium (Na) intake and an increase in potassium (K) intake can both independently reduce blood pressure (BP) [3–7]. This has led to the development of several specific nutrient recommendations in many countries [8–10]. However, people do not consume single nutrients in isolation, they eat foods usually in specific combinations or patterns, so that intakes of certain foods are negatively or positively correlated to certain other foods. Similarly, in food-based intervention studies that focus on changes in a single nutrient, it is very likely that changes in other nutrients will occur simultaneously. Investigating the effects of whole diets as consumed by population groups, rather than individual nutrients can provide additional insights into the relationship between dietary intakes and health.

Several studies have shown larger BP reductions using complex, multi-facetted dietary modifications rather than individual nutrient changes. For example, the landmark Dietary Approaches to Stop Hypertension (DASH) diet [11] which is rich in fruit, vegetables and low-fat dairy and low in fat, showed for the first time a greater BP lowering effect than single nutrient interventions and demonstrated the importance of focussing on overall dietary patterns in BP reduction. The clinically relevant falls in systolic BP (SBP) and diastolic BP (DBP) observed in normotensive (3.5 mmHg and 2.1 mmHg, respectively) and hypertensive (11.4 mmHg and 5.5 mmHg, respectively) individuals were larger than those previously observed in single nutrient intervention studies. Similarly a Mediterranean type dietary pattern has been shown to have a positive effect on blood pressure in several dietary intervention trials [12, 13]. Other dietary patterns observed to be inversely associated with blood pressure include a ‘vegetable dietary pattern’ in Japanese women [14], a ‘fruit and milk dietary pattern’ in Chinese men [15], ‘dairy and carbohydrate pattern’ in Korean adults [16], a ‘cosmopolitan dietary pattern’ in Dutch adults [17], a ‘vegetables and dairy pattern ‘or ‘ethnic foods and alcohol dietary pattern’ in British adults [18], and a ‘healthy dietary pattern’ in adult American Indians [19]. In addition, several dietary patterns have also been identified to be positively associated with blood pressure, including a ‘meat dietary pattern’ in Chinese men [15], a ‘traditional dietary pattern’ in Korean [16] and Dutch adults [17], and a ‘Western dietary pattern’ in Korean adults [16] and adult American Indians [19]. A recent systematic review and meta-analysis of the effect of dietary patterns on BP in adults, which included 5014 participants, found that the DASH, Mediterranean and Nordic diets all significantly reduced both SBP and DBP [20].

There is very little research examining the effect of dietary patterns on BP in the Australian context. Since foods and dietary patterns vary considerably between countries [21], country specific analysis is required. Dietary patterns also differ among certain populations within a country depending on factors such as age, sex and education level [22, 23]. To date there has been little published research on the association between dietary patterns and BP in Australian adults [24, 25]. A study by Livingstone et al, using data from two 24-h recalls on 4908 adults from the Australia National Nutrition and Physical Activity Survey, found a dietary pattern, determined by reduced rank regression, characterised by low fibre density and high sodium to potassium ratio (Na:K) and high saturated fat to polyunsaturated fat ratio, was associated with a higher prevalence of hypertension [26]. Therefore, the primary aim of this analysis was to investigate dietary patterns assessed by factor analysis, in a sample of free-living Australian adults and determine the association between dietary patterns and BP.

## Methods

### Study participants

Data for this analysis was drawn from the baseline or ‘usual’ reported dietary intake, prior to dietary modification, from volunteers who participated in a series of three dietary intervention studies conducted at Deakin University from 2002 to 2006. This is a pooled analysis of a series of related studies. Details of the intervention studies and main results of BP responses to dietary modifications have been reported previously [27–29].

Recruitment of participants to the intervention studies occurred via newspaper advertisements or BP screening sessions at shopping centres, Deakin University and workplaces. Eligible participants were over 25 years of age, had a screening BP of 120-160 mmHg systolic or 80-90 mmHg diastolic, were not diabetic, weighed < 150 kg, had not had a cardiovascular event in the last six months, ate their main meal outside the home less than twice per week, drank less than 30 standard (10 g alcohol) alcoholic drinks weekly, were not planning to change their smoking habits, were willing to cease taking any dietary supplements and were not pregnant, breastfeeding or trying to get pregnant. All participants provided written informed consent prior to commencing the studies. All studies and subsequent analysis were approved by the Deakin University Human Research Ethics Committee (EC47–2009).

### Blood pressure

#### Screening

Screening BP was measured using automated BP monitor (A&D Instruments, Oxon, UK). Participants were seated for all BP measurements. Mean screening BP was calculated using the last two of three measurements, taken at one-minute intervals following a two-minute rest period.

#### Home

Participants measured their home BP daily over a one-week period using an automated BP monitor (AND Model UA-767 for the 2002 study or AND Model UA-767-PC, A&D Co. Ltd., Tokyo, Japan) on their left arm, using a standard protocol for BP measurement [30]: the same time of day, same rest period and whilst seated. Detailed verbal and written instructions were provided. Three measurements were taken at one-minute intervals following a five-minute rest. Home BP measurements were either recorded by participants (UA-767 machines) or automatically recorded and stored on the machine and uploaded by study staff during the visit (UA-767-PC machines). The daily home BP was calculated as the mean of the last two of three measurements. The daily means were averaged over the week to determine the home BP for that week. Home blood pressure was the main outcome measure.

### Dietary assessment

Dietary intake was measured using two 24-h dietary recalls on random, non-consecutive days during the same week as the home blood pressure measurement. The 24-h dietary recalls were completed at the participant’s work site or study centre by a trained researcher on the day of the study appointment during an in-person interview. Food models were used to assist with portion size estimation. Participants were encouraged to keep recipes or labels from food products consumed and were encouraged to keep a record of what was eaten to assist with the 24-h recall.

Dietary data was entered and coded into a dietary analysis program to calculate mean daily nutrient intakes (FoodWorks, Professional Edition, Xyris Software, Queensland, Australia; Version 3.02 for 2002 and 2003 studies, and Version 4 for the remaining studies; using the standard Australian food composition database). The most recent database versions available at the time of data collection were NUTTAB 1995 [31] and AUSNUT 1999 [32]. Updated sodium values were added to AUSNUT 1999 in 2002 and used in this analysis. Any recipes obtained from participants were entered into the food composition database as a recipe and assigned to that participant. Any foods which were not already in the database were added using the nutrition information from the food label of the product. The mean of two 24-h dietary recalls completed at baseline by each participant were used in this analysis. Mean energy, sodium and potassium intakes were calculated from the two 24-h recalls and expressed as MJ/d and mmol/d respectively. The estimated sodium intake did not include any allowance for discretionary salt intake (i.e. salt added at the table or in cooking).

All food and drinks were assigned to food groups on the basis of the food grouping system developed by Food Standards Australia New Zealand (FSANZ) which has 20 major food groups. According to the similarities in food nutrient profiles, several major food groups were combined or spilt based on key nutrients such as sodium, fibre or fat. For example the major food group ‘Milk products and dishes’ was split into four food sub-groups namely Cheese; Milk and yoghurt– high fat (> 1%); Milk and yoghurt– low fat (< 1%); and Soy milk & Flavoured milk. Dishes were a combination of ingredients/foods to make a meal/dish as opposed to an individual foods. For example mixed cereal dishes included hamburgers or sandwiches, meat/poultry dishes included stews or curries or stir fry, eggs dishes included quiche or scrambled eggs, vegetable dishes included cauliflower cheese, pasta and rice dishes included cheese and spinach ravioli or Asian noodle dishes. Dietary patterns were based on 34 final food sub-groups. Additional file 1: Table S1 shows the 34 food sub-groups used in the analysis (see Additional file 1).

### Measurement of anthropometry and other variables

Body weight was measured to the nearest 0.05 kg on digital scales (UC-321 Precision Personal Health Scale, A&D Weighing, Australia) with participants wearing light clothing and no shoes. Height was measured to the nearest 0.1 cm using a stadiometer (Portable Height Scale, Mentone Educational, Australia). Body mass index (BMI) was calculated from measured height (ht) and weight (wt) using the following formula: BMI (kg/m^2^) = wt (kg) / ht. (m)^2^.

All participants completed self-administered questionnaires which covered demographic information (age and sex), alcohol intake, medication use, education level and physical activity. Smoking status was defined as current smoker or non-smoker. Education level was defined by the highest level of schooling attained. Participants were asked to quantify the number of hours of vigorous physical activity they did per week. In another question, they were also asked to self-assess their overall physical activity level. A self-report of more than 4 h OR a self-assessment of quite/very/extremely active was considered to be physically active for the purpose of this study. For the purpose of this analysis, being physically active was defined as either a self-report of more than four hours per week of vigorous physical activity or a self-report as either quite active, very active or extremely active.

### Statistical analysis

Dietary patterns were derived from the 24-h recall data using factor analysis with principal component analysis extraction and varimax rotation based on weight of food consumed (grams). Twenty food groups were entered in to the analysis which is appropriate given statistical recommendations for the ratio of variables used and number of participants in the study [33, 34], and comparisons with previous studies in the area [22, 35, 36]. The number of dietary patterns identified was determined based on eigenvalues > 1.25, followed by the identification of a break in the scree plot and interpretation as per Schulze et al. [37, 38] Nine factors had a eigenvalue > 1.25, however examination of the scree plot revealed that the eigenvalues of the factors decreased substantially after third factor and then remained similar indicating that three factors would be optimal.

Items with an absolute factor loading of 0.20 or more were considered to load on a factor and thus retained in the dietary pattern score calculation [37, 39]. Dietary pattern scores were calculated using the weight of each food item and the weighting determined by the factor analysis. Food items with absolute factor loadings of < 0.20 were not considered to significantly contribute to a pattern and thus were not included in the dietary pattern score calculation. If a particular food item loaded highly on more than one factor (cross-loading), they were retained only in the dietary pattern in which their factor loading was highest unless the direction of the factor loading was opposite.

To address the primary aim, linear regression analysis was used to explore the association between dietary pattern score and BP. Models were adjusted for age, gender and BMI (model 1) which are known predictors of BP. The models were also adjusted for use of anti-hypertensive medication, smoking status, physical activity and education level (model 2). The final model was additionally adjusted for energy intake to determine if the effects were independent of total energy intake (model 3). Additionally, dietary pattern scores were categorised according to tertiles with tertile 1 corresponding to the lowest tertile of dietary pattern score. Mean dietary pattern scores were determined for each tertile. Associations between dietary pattern scores and categorical variables (gender and lifestyle factors such as anti-hypertensive medication use, physical activity, smoking status, and education level) were assessed using Chi-square analysis. Associations between dietary pattern scores and continuous variables (age and BMI) and were calculated using one-way between groups analysis of variance. Mean nutrient intakes (mean of two 24-h recalls) for each tertile of dietary pattern score were calculated and linear trends were estimated using one-way between groups analysis of variance.

Data were analysed using SPSS for WINDOWS (version 17.0; SPSS Inc., Chicago, IL, USA) and Statistical Analysis Systems statistical software (version 9.1; SAS Institute, NC, USA). Values are presented as means and SD. *P*-values < 0.05 were considered significant.

## Results

Of the 344 eligible participants who attended baseline appointments, 251 (73%) had two complete 24-h dietary recalls at baseline and were included in this analysis. The characteristics of all participants are shown in Table 1. The age of the sample ranged from 28 to 81 years. The male participants were younger, taller and heavier than the females, but there was no difference in BMI (Tale 1).

**Table 1:** Participant characteristics (*n* = 251)

	All	Females (*n* = 139)	Males (*n* = 112)
Age (years)	55.1 (9.1)	58.0 (6.4)***	51.5 (0.6)
Height (cm)	168.3 (9.1)	162.7 (6.9)***	175.3 (6.0)
Weight (kg)	83.8 (13.7)	78.2 (12.3)***	90.8 (12.0)
BMI (kg/m^2^)	29.5 (3.9)	29.5 (4.4)	29.5 (3.1)
Home blood pressure (mmHg)			
Systolic	128.8 (11.9)	127. 3 (11.7)*	130.6 (12.0)
Diastolic	81.3 (8.4)	80.3 (8.4)	82.4 (8.3)
% On anti-hypertensive medication ^	38.6	40.3	36.6
% physically active	25.4	23.0	28.6
% current smoker	3.6	2.9	4.5
Education level – % high school or lower	33.9	46.8	17.9
Energy intake (kJ/day)	9177.8 (3109.3)	7573.4 (2027.0)***	11,168.8 (3072.8)
Sodium intake (mmol/day)	119.1 (49.4)	100.0 (37.5)***	142.7 (52.2)
Energy adjusted sodium intake (mmol/MJ)	13.1 (3.9)	13.4 (4.2)	12.9 (3.5)
Potassium intake (mmol/day)	91.3 (27.4)	80.0 (22.5)***	105.3 (26.4)
Energy adjusted potassium intake (mmol/MJ)	10.4 (2.8)	10.9 (3.0)***	9.7 (2.3)
Sodium: Potassium molar ratio	1.36 (0.54)	1.32 (0.53)	1.40 (0.55)

*All data are mean (SD) or percentage of participants; Abbreviations: BMI, body mass index.*

Difference between males and females *P <  0.05, **P <  0.01, ***P <  0.001.

^ *39% of the sample on anti-hypertensive medication is consistent with the incidence of hypertension in the adult community (~ 34% of Australians aged 18 and over have high blood pressure, based on measured data from the 2017–18 Australian Bureau of Statistics National Health Survey, and this increases with age). Participants also needed to have been on the AHT medication for enough time (screening question) that their blood pressure was stable.*

Three major dietary patterns were identified which explained 18.2% (7.2, 5.7 and 5.3%, respectively) of the total variation in food intake among individuals in this sample (Table 2).

**Table 2:** The three identified dietary patterns in a sample of Australian adults (*n* = 251)

Dietary Pattern 1	Factor loading^*1*^	Dietary Pattern 2	Factor loading^*1*^	Dietary Pattern 3	Factor loading^*1*^
Fruit drinks, cordial^a^ & soft drinks	0.65	Bread – low fibre (white)	0.50	Breakfast cereals – high sodium	0.62
Processed meat	0.53	Pasta, noodles &rice	0.49	Milk & yoghurt – high fat (> 1%)	0.51
Fried potatoes	0.48	Meat, egg & poultry dishes	0.47	Take-away^b^	0.44
Alcoholic beverages	0.45	Mixed cereal dishes	0.26	Pasta & rice dishes	0.32
Meats, poultry & egg	0.43	Seeds & nuts ^	0.25	Fruit juices	0.28
Sauces & salad dressings	0.41	Vegetable dishes	0.20	Soy milk & flavoured milk	0.25
Fats & oils	0.35	Fried potatoes	−0.25	Fruit	0.23
Cheese	0.22	Milk & yoghurt – low fat (< 1%)	− 0.30	Bread – low fibre (white)	− 0.21
Breakfast cereals – low sodium	−0.23	Meats, poultry & egg	−0.31	Vegemite^c^	−0.24
Canned fish & fish dishes	−0.33	Vegetable juices	−0.33	Vegetable dishes	−0.24
Tea & coffee	−0.40	Vegetables	−0.43	Cheese	−0.36
		Bread – high fibre	−0.48	Snacks	−0.36
Variance Explained (%)	7.2		5.7		5.3

^*1*^*Absolute values <  0.2 are not shown;* ^*a*^*cordial is a non-alcoholic fruit drink concentrate;* ^*b*^ *take-away includes fast foods such as dim sums, pizza, spring rolls;* ^*c*^*Vegemite is an Australian food spread made from brewers’ yeast extract. The following 4 food groups are not included in any dietary pattern:* *Mineral & electrolyte drinks,* *Fish & seafood,* *Soup, and* *Cakes & sweets as their absolute factor loading was <  0.2 and thus were not considered to significantly contribute to a pattern. ^The food group seeds & nuts consisted mainly of salted nuts in this population group*

Dietary Pattern 1 was characterised by a high consumption of fruit drinks and soft drinks, processed meat, fried potatoes, alcoholic beverages, meats, poultry and egg, sauces and salad dressings, fats and oils and cheese, and a low consumption of tea and coffee, canned fish and fish dishes and low-sodium breakfast cereals (Table 2). Dietary pattern 2 consisted of a high consumption of low-fibre bread, past and rice, dishes containing meat, poultry and egg, mixed cereal dishes, seeds and nuts (which were mainly salted nuts in this population group) and vegetable dishes, and low consumption of high-fibre bread, vegetables, vegetable juices, meat, poultry and egg, low-fat milk and yoghurt and fried potatoes (Table 2). Finally, dietary pattern 3 was characterised by high consumption of high-sodium breakfast cereals, high fat milk and yoghurt, takeaway, pasta and rice dishes, fruit juices, soy milk and flavoured milk and fruit, and low consumption of snacks, cheese, vegetable dishes, Vegemite and low-fibre bread (Table 2). Additional file 2: Table S2 shows the full factor loading matrix for the three dietary patterns (see Additional file 2).

There was a significant positive association between the ‘Dietary Pattern 2’ and home SBP which remained after adjustment for age, gender, BMI, use of anti-hypertensive medication, smoking status, physical activity, education level and energy intake (model 3) (Table 3). In model 3, each unit increase in consumption of Dietary Pattern 2 was associated with a 1.88 mmHg increase in home SBP (Table 3). This association remained when additionally adjusted for alcohol (data not shown). Dietary Pattern 1 and Dietary Pattern 3 were not associated with home SBP after adjusting for potential confounders in all models. There were no significant associations with home DBP and any of the dietary patterns.

**Table 3:** Association between dietary pattern scores and home blood pressure (*n* = 251)

	Home SBP			Home DBP		
	β	95% CI	*P*-value	β	95% CI	*P*-value
Dietary Pattern 1						
Model 1	0.10	(−1.76, 1.96)	0.916	−0.63	(−1.98, 0.72)	0.361
Model 2	0.12	(−1.82, 2.05)	0.903	−0.61	(−2.03, 0.80)	0.394
Model 3	0.41	(−1.69, 2.50)	0.704	−0.45	(−1.99, 1.08)	0.561
Dietary Pattern 2						
Model 1	1.78	(0.09, 3.46)	**0.039**	1.14	(−0.09, 2.37)	0.069
Model 2	1.81	(0.10, 3.52)	**0.038**	1.08	(−0.18, 2.33)	0.091
Model 3	1.88	(0.16, 3.60)	**0.032**	1.14	(−0.12, 2.41)	0.075
Dietary Pattern 3						
Model 1	−0.45	(−2.06, 1.16)	0.585	−1.10	(−2.26, 0.06)	0.064
Model 2	−0.32	(−1.96, 1.32)	0.701	−1.06	(−2.26, 0.13)	0.080
Model 3	−0.25	(−1.91, 1.41)	0.765	−1.01	(−2.22, 0.19)	0.100

Significant data are in bold

Model 1 = Adjusted for age, gender and BMI.

Model 2 = Model 1 + anti-hypertensive medication use, smoking status, physical activity, education.

Model 3 = Model 2 + energy intake.

Abbreviations: SBP, systolic blood pressure. DBP, diastolic blood pressure

The three dietary patterns were associated with a number of participant characteristics (Table 4).

**Table 4:** Participant characteristics according to tertiles of dietary pattern score (*n* = 251)

	Dietary Pattern 1				Dietary Pattern 2				Dietary Pattern 3			
	Tertile 1	Tertile 2	Tertile 3	P- trend^1^	Tertile 1	Tertile 2	Tertile 3	Ptrend^1^	Tertile 1	Tertile 2	Tertile 3	Ptrend^1^
Dietary Pattern Score^*2*^	***−0.78 (−2.3 to − 0.37)***	***−0.12 (− 0.36 to 0.15)***	***0.91 (0.16 to 4.0)***		***−0.90 (− 2.8 to − 0.36)***	***−0.06 (− 0.35 to 0.31)***	***0.96 (0.32 to 2.84)***		***−0.89 (− 2.9 to − 0.39)***	***−0.76 (− 0.39 to 0.28)***	***0.97 (0.29 to 3.85)***	
Age (y)	56.2 (54.3, 58.2)	54.6 (52.7, 56.6)	54.4 (52.4, 56.3)	0.345	57.6 (55.6, 59.5)	55.1 (53.1, 57.0)	52.6 (50.7, 54.5)	**0.003**	53.6 (51.6, 55.5)	56.7 (54.8, 58.7)	55.0 (53.0, 56.9)	0.080
BMI (kg/m^2^)	28.6 (27.8, 29.4)	29.9 (29.1, 30.7)	30.1 (29.3, 30.9)	**0.022**	29.8 (29.0, 30.6)	29.8 (29.0, 30.7)	29.0 (28.1, 29.8)	0.214	29.7 (28.8, 30.5)	29.8 (29.0, 30.6)	29.1 (28.3, 30.0)	0.496
% Male	23.8	41.7	68.7	**< 0.001**	39.3	36.1	58.3	**0.016**	45.2	34.9	53.6	0.314
% Taking AHT	33.3	32.1	50.6	**0.026**	40.5	34.9	40.5	1.000	44.0	36.1	35.7	0.304
% Physically active*	21.7	26.2	28.9	0.329	28.6	19.3	28.9	1.000	26.5	26.5	23.8	0.724
% Current smoker*	0.0	3.6	7.3	**0.011**	4.8	2.4	3.6	0.838	6.0	4.8	0.0	0.059
% Education – High school or lower	34.5	35.7	31.3	0.684	35.7	28.9	36.9	0.935	34.5	38.6	28.6	0.464

Significant data are in bold

^1^ Difference in proportions computed by Chi-square analysis (categorical variables) or linear trends computed by analysis of variance (continuous variables) across tertiles ^2^Mean (range) dietary pattern score

*n = 250

All other data are mean (95% CI) or percentage of participants,

Abbreviations: BMI body mass index; AHT anti-hypertensive medication

Dietary Pattern 2 was inversely associated with age and a greater percentage of males. There were no associations between Dietary Pattern 2 and BMI, anti-hypertensive medication use, physical activity, smoking status or education level. Dietary Pattern 1 was positively associated with BMI, a greater percentage of males, anti-hypertensive medication use, and a greater percentage of smokers. Dietary Pattern 3 was not associated with any lifestyle factors investigated.

To further investigate the nutrient composition of each dietary pattern, linear correlation and oneway between groups analysis of variance analyses were performed. Dietary Pattern 2 score was positively correlated with dietary sodium (r = 0.476, *P* = 0.001) and the sodium to potassium molar ratio (r = 0.311, *P* = 0.000) and inversely correlated with potassium (r = − 0.160, *P* = 0.011). Dietary.

Pattern 1 score was positively correlated with sodium (r = 0.476, *P* = 0.000), potassium (r = 0.394, *P* = 0.000) and the sodium to potassium molar ratio (r = 0.180, *P* = 0.004). Dietary Pattern 3 score was positively correlated with potassium (r = 0.326, *P* = 0.000) and inversely correlated with the sodium to potassium molar ratio (r = − 0.271, *P* = 0.000). Correlations with other individual nutrients were not assessed.

Similar results were found when the data were split into tertiles of dietary pattern score (Table 5).

**Table 5:** Mean nutrient intakes for each tertile of dietary pattern score (n = 251)

	Dietary Pattern 1				Dietary Pattern 2				Dietary Pattern 3			
	Tertile 1	Tertile 2	Tertile 3	P-trend^1^	Tertile 1	Tertile 2	Tertile 3	P-trend^1^	Tertile 1	Tertile 2	Tertile 3	P-trend^1^
Dietary Pattern Score^*2*^	***−0.78 (−2.3 to − 0.37)***	***−0.12 (− 0.36 to 0.15)***	***0.91 (0.16 to 4.0)***		***−0.90 (− 2.8 to − 0.36)***	***− 0.06 (− 0.35 to 0.31)***	***0.96 (0.32 to 2.84)***		***− 0.89 (− 2.9 to − 0.39)***	***−0.08 (− 0.39 to 0.28)***	***0.97 (0.29 to 3.85)***	
E intake (MJ/day)	7.8 (7.2, 8.3)	8.6 (8.0, 9.1)	11.3 (10.7, 11.8)	**< 0.001**	9.1 (8.4, 9.8)	8.4 (7.8, 9.1)	10.0 (9.4, 10.7)	**0.002**	9.0 (8.4, 9.7)	8.9 (8.3, 9.6)	9.6 (8.9, 10.2)	0.342
Na intake (mmol/day)	99.8 (90.2, 109.4)	108.4 (98.8, 118.0)	149.3 (140.1, 159.4)	**< 0.001**	117.6 (106.8, 128.3)	107.5 (97.3, 118.0)	131.9 (121.7, 142.4)	**0.005**	125.1 (114.7, 135.8)	115.3 (104.8, 126.1)	116.7 (106.1, 127.4)	0.380
E adjusted Na (mmol/d)	115.9 (109.4, 122.3)	115.5 (108.8, 122.1)	125.9 (116.7, 135.1)	0.088	118.5 (111.3, 125.6)	116.2 (109.6, 122.7)	122.5 (113.7, 131.3)	0.487	126.8 (119.5, 134.2)	118.1 (110.4, 125.9)	112.2 (105.0, 119.4)	**0.022**
K intake (mmol/day)	79.0 (73.7, 84.4)	89.4 (84.0, 94.8)	105.7 (100.2, 111.1)	**< 0.001**	99.8 (94.1, 105.6)	87.0 (81.2, 92.8)	87.1 (81.4, 92.9)	**0.002**	82.2 (76.5, 87.8)	91.5 (85.8, 97.2)	100.3 (94.6, 106.0)	**< 0.001**
E adjusted K (mmol/d)	87.4 (83.3, 91.5)	93.1 (89.0, 97.2)	93.4 (88.5, 98.4)	0.096	100.3 (95.3, 105.2)	91.5 (88.0, 95.0)	82.2 (78.5, 85.9)	**< 0.001**	83.1 (79.0, 87.1)	93.0 (88.5, 97.5)	97.9 (93.9, 102.0)	**< 0.001**
Na:K molar ratio	1.33 (1.22, 1.45)	1.25 (1.14, 1.37)	1.49 (1.37, 1.60)	**0.016**	1.24 (1.12, 1.35)	1.27 (1.15, 1.38)	1.57 (1.46, 1.68)	**< 0.001**	1.59 (1.48, 1.70)	1.30 (1.19, 1.41)	1.18 (1.07, 1.29)	**< 0.001**

Significant data are in bold

^1^P-trend computed by one-way between groups analysis of variance (ANOVA)

^2^Mean (range) dietary pattern score

All other values are mean (95% CI) for each tertile

Abbreviations: E energy; Na sodium; K potassium; Na:K

The mean nutrient intakes for each tertile of dietary pattern score are shown in Table 5. Dietary Pattern 2 was positively associated with intakes of energy, sodium and the sodium to potassium molar ratio and inversely associated with potassium. After adjusting for energy, Dietary Pattern 2 was no longer associated with sodium but remained inversely associated with potassium (Table 5). Dietary Pattern 1 was positively associated with intakes of energy, sodium, potassium and the sodium to potassium molar ratio but the associations with sodium and potassium no longer remained after adjustment for energy intake. Dietary Pattern 3 was positively associated with potassium intake and inversely associated with the sodium to potassium molar ratio. After adjustment for energy, Dietary Pattern 3 remained positively associated with potassium and the inverse association with sodium became significant (Table 5).

## Discussion

In this sample of free-living Australian adults we identified three dietary patterns. We found that a Dietary Pattern 2 was positively associated with home SBP. This finding was independent of potential confounding factors such as age, gender, BMI, anti-hypertensive medication use, physical activity, smoking status, education level and energy intake. Each unit increase in Dietary Pattern 2 score in this adjusted model was associated with a 2 mmHg higher home SBP. On a population level this relatively small difference in blood pressure is significant, as a 2 mmHg reduction in the mean SBP of a population has been predicted to result in a 5% reduction in 16-year CVD mortality [40]. Similarly, large population studies have estimated a 2–4% increase in relative risk of death due to CVD, for every 1 mmHg increase in SBP [41–44].

Whilst some comparisons can be made with other Australian studies, it is still difficult to directly compare our results with other studies, even when they have used the same factor analysis method of dietary pattern analysis. This is because there are often differences in the number and composition of food groupings used as well as differences in the method of dietary data collection (e.g. 24-h recall vs food frequency questionnaire). Comparison with international data can also be challenging due to the different patterns identified, the different population groups analysed and the different methodologies used. However some similarities can be drawn with the current analysis. For example, in a sample of Chinese men [15] a dietary pattern characterised by a high intake of meat, was positively associated with DBP, independent of lifestyle and socioeconomic factors. Data from Korea found ‘Western pattern’, high in ham, fast food, fats and oils, carbonated beverages, noodles, meat, and alcohol was positively associated with both SBP and DBP. In the Netherlands, van Dam et al [17] found a positive association between SBP and a ‘traditional diet’ characterised by meat, potatoes, coffee, beer and eggs. A study of American Indian men found a ‘Western diet’, which consisted of a higher consumption of fast food, snack chips, fried potatoes, prepared main dishes, sweet beverages and animal fats, was associated with a higher SBP, after adjusting for confounders [19]. Whilst it is difficult to directly compare, there are some similarities between these other previously described dietary patterns which have shown associations with BP, and our Dietary Pattern 2, which was also positively associated with home SBP, namely a high intake of meat, fast food (take-away), animal fats and prepared main dishes (mixed dishes).

In the current study, Dietary Pattern 2 had a high factor loading for ‘mixed dishes’ (meat, poultry and egg dishes; mixed cereal dishes; and vegetable dishes) which often involve the use of ready-made sauces or packet flavour mixes which are usually very high in sodium (up to ~ 3000 mg/100 g). We have shown previously [45] that these mixed dishes (stews, curries, stir fries) make a significant contribution to the total sodium content in the Australian diet. As well as these mixed dishes, Dietary Pattern 2 was also characterised by a high consumption of high-sodium foods such as white bread, and was low in high-potassium foods such as high-fibre bread, vegetables, vegetable juices, low-fat milk and yoghurt and fried potato. Dietary Pattern 2 score was positively correlated with sodium but also with energy. A higher intake of food (and thus energy) leads to an increase intake in nutrients [46] and we have shown previously that higher energy intakes correlate with higher sodium and potassium intakes [45]. Thus to assess whether the association with sodium was driven by energy, we adjusted for energy intake and found there was no longer an association between Dietary Pattern 2 score and energy-adjusted sodium. However, energy-adjusted potassium remained inversely correlated to Dietary Pattern 2 score. These associations point to Dietary Pattern 2 being high in foods that are high in both energy and sodium but also, potentially more importantly, low in potassium containing foods.

Dietary Pattern 1 in the current study, was characterised by energy-dense, nutrient-poor foods and is similar to the Western dietary pattern described by other researchers [16, 19]. While this pattern is also associated with higher sodium and higher energy, we did not observe any relationship with BP. This may be due to this pattern also being associated with higher potassium, resulting in a more moderate sodium to potassium molar ratio than Dietary Pattern 2. A high potassium intake has been shown to negate some of the negative effects of a high sodium intake [47] and it has been consistently demonstrated that sodium to potassium molar ratio of an individual’s diet is more strongly and consistently related to BP [48] and CVD risk [49] than the sodium level alone. The sodium to potassium molar ratio was more strongly correlated with Dietary Pattern 2 than Dietary Pattern 1. However, Dietary Pattern 1 was also positively associated with energy intake and there were no significant associations with either sodium or potassium once we adjusted for energy. This is likely because the main sources of potassium in this dietary pattern are fried potatoes, meat and fruit drinks, which are also likely high in energy. Consuming more of these foods leads to a higher potassium intake but also a higher energy intake. We have previously shown potatoes to be the main food source of potassium in the Australian diet providing 8–10% of the daily potassium intake [45]. However, given that this pattern loaded highly on fried potatoes specifically, it potentially involves additional salt and fat which will increase the sodium and energy content of the diet and decrease the overall nutritional quality.

In contrast to Dietary Pattern 2, Dietary Pattern 3 contained numerous high-potassium foods such as breakfast cereals, milk and yoghurt, fruit juices, soy milk and fruit and was low in high-sodium foods such as snacks, cheese, vegetable dishes, vegemite and white bread. Although there was some indication that this diet was inversely associated with home DBP, this was not significant and there was no association with home SBP. Dietary Pattern 3 was the only dietary pattern where both the sodium association and potassium association remained after adjusting for energy. There was an inverse association with energy adjusted sodium and a positive association with energy adjusted potassium but still no significant effect on BP.

When dietary indexes or dietary scores are used to assess overall diet quality as a measure of healthy eating patterns, female gender, older age and higher income or education level have been associated with better diet scores [22]. We found no association with education for any of the dietary patterns but this may be because overall we had a well-educated population and did not have a wide range of education levels. We did show an association with gender, with males being more likely to consume Dietary Pattern 1 and Dietary Pattern 2 which had the least favourable sodium, potassium and sodium to potassium molar ratio profiles and thus are more likely to have negative effects on BP. Dietary Pattern 2 was also inversely associated with age. Our results are consistent with previous research which has also shown an association between the consumption of convenience meals and gender and age, with men and younger adults being more likely to consume ready-meals [50]. Dietary Pattern 1 was associated with several lifestyle factors known to increase CVD risk. Those on antihypertensive medication (i.e. those with hypertension), smokers and those with a higher BMI were more likely to consume dietary pattern 1, which may indicate a low level of health consciousness or poor health choices.

Using data-driven statistical approaches to determine the major dietary patterns of a population and examine the effects of these patterns on specific outcome measures or risk factors is a common approach [22, 33, 34, 37]. A key strength of this type of analysis is that it can identify the existing patterns of dietary behaviour within a population. Therefore, any dietary recommendations that are subsequently made are based on dietary patterns that already exist within a population and are thus likely to be better accepted and potentially more successful in achieving dietary change. Furthermore, the WHO now recommends the use of food based dietary guidelines, instead of nutrient population goals [51].

The selection or creation of the food groups used is critical in exploratory factor analysis, therefore the food groups we created were based key nutrients for BP, our health outcome of interest, which may be considered a strength over using generic food groups. However the variation in both the type and proportion of individual foods within broader food groups, needs to be considered in dietary pattern analysis and interpretation. We used two 24-h recalls for our dietary assessment which may not be considered reflective of usual intake, but one or two days’ worth of recall data have been used previously in dietary pattern analysis [52, 53] as recall data provides a more accurate assessment of dietary intake than food-frequency data [54] in this type of analysis. Dietary patterns are strongly correlated with several other lifestyle factors, although we did control for the key factors known to affect BP (age, sex, BMI, anti-hypertensive use, smoking, physical activity). The study population used in this analysis may not be representative of the Australian population since this group volunteered to participate in dietary intervention studies and therefore these results can not necessarily be generalised to the Australian population. It is likely that the dietary intake of our sample is more homogenous than that of a of the general population as the selection criteria excluded those who ate their main meal outside the home more than twice a week and those who drank more than 30 standard drinks per week. Thus with less variation in dietary intake, there may be less ability to detect associations with BP. Although our sample size was less than 300 individuals, this is comparable to other analyses assessing associations between dietary patterns and other markers of chronic disease [24, 25, 36, 55, 56]. A key strength of this study is the use of home BP measurements which have been shown to share the many advantages of ambulatory monitoring. Measuring BP at home more closely reflects real life conditions and as such is more reproducible and more able to predict hypertensive organ damage. There is also no ‘white-coat’ effect with home BP monitoring [57]. In addition, this study assessed the dietary intake of free-living men and women, on two non-consecutive days.

## Conclusion

In this study a dietary pattern characterised by higher intake of white bread, pasta noodles and rice, mixed dishes and salted nuts, and lower intake of low-fat milk and yoghurt, vegetable juices, vegetables, high-fibre bread, meat, poultry and eggs and fried potatoes was associated with a higher systolic BP. Furthermore, this dietary pattern was associated with a lower intake of potassium and a higher intake of energy which increases the sodium content, both known to be key risk factors for CVD [3–5, 58]. Our results lend support to the current recommendations to increase intakes of high potassium, low energy foods such as fruits and vegetables and low fat dairy products, for cardiovascular health. The Australian Dietary Guidelines also promote a dietary pattern high in foods from the fruit and vegetables and reduced-fat dairy food groups, and recommend limiting foods containing added salt. Further studies in larger samples of varying dietary intakes would be able to confirm these important results.

## Supplementary information

**Additional file 1: Table S1.** The 34 food sub-groups used in the analysis. For dietary pattern analysis, all food and drinks were assigned to food groups on the basis of the food grouping system developed by Food Standards Australia New Zealand (FSANZ) which has 20 major food groups. According to similarities in food nutrient profiles, several major food groups were combined or spilt based on key nutrients resulting in 34 sub food groups. This table shows the final 34 food sub groups used in dietary pattern analysis.

**Additional file 2: Table S2.** Factor loading matrix for the three dietary patterns identified in this sample of Australian adults (*n* = 251). Table S2 shows the full factor loading matrix (for values > 0.2) for each of the three dietary patterns for all of the 34 food groups, including those food groups which don’t load on any pattern.

## Data Availability

The datasets used and/or analysed during the current study are available from the corresponding author on reasonable request.

## Abbreviations

AHT: Anti-hypertensive medication
BMI: Body mass index
BP: Blood pressure
CVD: Cardiovascular disease
DASH: Dietary Approaches to Stop Hypertension
DBP: Diastolic blood pressure
E: Energy
FSANZ: Food Standards Australia New Zealand
K: Potassium
Na: Sodium
Na:K: Sodium to potassium ratio
SBP: Systolic blood pressure
