# Supplementary material for: Associations between dietary patterns and blood pressure in a sample of Australian adults
Source: Nutr J. 2020 Jan 14;19:5. doi: 10.1186/s12937-019-0519-2 (PMC6961350; doi:10.1186/s12937-019-0519-2)
Supplement: Supplementary file 1 — Additional file 1: Table S1. The 34 food sub-groups used in the analysis. For dietary pattern analysis, all food and drinks were assigned to food groups on the basis of the food grouping system developed by Food Standards Australia New Zealand (FSANZ) which has 20 major food groups. According to similarities in food nutrient profiles, several major food groups were combined or spilt based on key nutrients resulting in 34 sub food groups. This table shows the final 34 food sub groups used in dietary pattern analysis. [file 12937_2019_519_MOESM1_ESM.docx]

**Table S1 - The 34 food sub-groups used in the analysis**

| **Food sub-group** | **Food items** |
| --- | --- |
| Tea and coffee | Tea  Coffee and coffee substitutes |
| Vegetable juices | Vegetable juices |
| Fruit juices | Single fruit juices  Mixed fruit juices |
| Fruit drinks, cordials and soft drinks | Fruit drinks and cordials  Soft drinks |
| Mineral & electrolyte drinks | Flavoured mineral waters  Electrolyte drinks  Electrolyte drink bases  Mineral water, soda water |
| Bread - low fibre (white) | Regular breads, and rolls (white)  Fancy breads, flat breads, English-style muffins and Crumpets |
| Bread - high fibre | Regular breads, and rolls (mixed grain, wholemeal, rye, high-fibre white) |
| BF cereals - high sodium |  |
| BF cereals - low sodium | Breakfast cereal, bran, unprocessed  Breakfast cereal, muesli, untoasted  Breakfast cereal, hot porridge type |
| Pasta, noodles and rice | Flours and other cereal grains and starches  Pasta and egg noodles  Rice  Mature legumes and pulses |
| Pasta and rice dishes | Filled pasta, ravioli  Noodles, Asian style  Rice products  Flavoured rice |
| Mixed cereal dishes | Sandwiches  Filled rolls and hamburgers  Savoury pasta and sauce dishes  Savoury rice-based dishes  Mature legume and pulse products and dishes |
| Take-away | Pizza  Taco and tortilla-based dishes  Chiko-type' rolls, dim sims and spring rolls  Fried and battered Fish and seafood products (calamari, prawns, scallops) |
| Fats and oils | Dairy fats  Margarine & Vegetable oil  Other fats & Unspecified fats  Cream |
| Fish and seafood | Fin fish (excluding canned)  Crustacea and molluscs (excluding canned)  Other sea and freshwater foods |
| Canned fish and fish dishes | Packed (canned and bottled) fish and seafood  Mixed dishes with fish or seafood as the major component |
| Fruit | Pome fruit  Berry fruit  Citrus fruit  Stone fruit  Tropical fruit  Other fruit  Mixtures of two or more groups of fruit  Dried fruit, preserved fruit  Mixed dishes where fruit is the major component |
| Meats & poultry & egg | Eggs  Muscle meat (beef, pork, lamb, veal)  Game and other carcase meats  Poultry and feathered game |
| Processed meat | Bacon  Ham  Sausages, frankfurts and saveloys  Processed meat  Mixed dishes where lamb or pork, bacon, ham is the major component |
| Mixed meat, egg and poultry dishes | Dishes where egg is the major ingredient  Organ meats and offal, products and dishes  Mixed dishes where beef or veal is the major component  Mixed dishes where lamb or pork, bacon, ham is the major component  Mixed dishes where poultry or game is the major component |
| Dairy milk & yoghurt (> 1% fat) | Dairy milk  Yoghurt  Frozen milk products  Other dishes where milk or a milk product is the major component (custard, dairy desserts) |
| Soy milk & flavoured milk | Milk substitutes  Flavoured milks |
| Dairy milk & yoghurt (< 1% fat) |  |
| Cheese |  |
| Soup | Soup  Dry soup mix  Canned condensed soup |
| Seeds & nuts | Seeds and seed products  Nuts and nut products |
| Sauces & dressings | Gravies and savoury sauces  Pickles, chutneys and relishes  Salad dressings  Herbs, spices, seasonings and stock cubes |
| Fried potatoes | Potatoes, chips, wedge, hash brown  Potato products |
| Vegetables |  |
| Vegetable dishes | Potato dishes  Dishes where vegetable is the major component |
| Snacks (Corn, extruded, pretzels, etc.) | Savoury biscuits  Snack foods  Potato snacks  Corn snacks  Extruded snacks  Pretzels and other snacks |
| Cakes and sweets | Sweet biscuits  Cakes, buns, muffins, scones, cake-type desserts  Pastries  Batter-based products  Sugar products and dishes  Sugar, honey and syrups |
| Alcoholic beverages | Beers  Wines  Spirits  Other alcoholic beverages |
| Vegemite |  |
