# Supplementary material for: Associations between dietary patterns and blood pressure in a sample of Australian adults
Source: Nutr J. 2020 Jan 14;19:5. doi: 10.1186/s12937-019-0519-2 (PMC6961350; doi:10.1186/s12937-019-0519-2)
Supplement: Supplementary file 2 — Additional file 2: Table S2. Factor loading matrix for the three dietary patterns identified in this sample of Australian adults (n = 251). Table S2 shows the full factor loading matrix (for values > 0.2) for each of the three dietary patterns for all of the 34 food groups, including those food groups which don’t load on any pattern. [file 12937_2019_519_MOESM2_ESM.docx]

**Table S2- Factor loading matrix for the three dietary patterns identified in this sample of Australian adults (n=251)**

| **Food group** | **Dietary Pattern 1** | **Dietary Pattern 2** | **Dietary Pattern 3** |
| --- | --- | --- | --- |
| Tea and coffee | -0.40 |  |  |
| Vegetable juices |  | -0.33 |  |
| Fruit juices |  |  | 0.28 |
| Fruit drinks, cordial & soft drinks | 0.65 |  |  |
| Mineral & Electrolyte drinks |  |  |  |
| Bread – low fibre (white) |  | 0.50 | -0.21 |
| Bread - high fibre |  | -0.48 |  |
| Breakfast cereals |  |  | 0.62 |
| Breakfast cereals – low sodium | -0.23 |  |  |
| Pasta, noodles and rice |  | 0.49 |  |
| Pasta & rice dishes |  |  | 0.32 |
| Mixed cereal dishes |  | 0.26 |  |
| Take-away |  |  | 0.44 |
| Fats and oils | 0.35 |  |  |
| Fish & seafood |  |  |  |
| Canned fish & fish dishes | -0.33 |  |  |
| Fruit |  |  | 0.23 |
| Meats, poultry and egg | 0.43 | -0.31 |  |
| Processed meat | 0.53 |  |  |
| Meat, egg & poultry dishes |  | 0.47 |  |
| Milk & yoghurt – high fat (>1%) |  |  | 0.51 |
| Soy milk & flavoured milk |  |  | 0.25 |
| Milk & yoghurt – low fat (<1%) |  | -0.30 |  |
| Cheese | 0.22 |  | 0.36 |
| Soup |  |  |  |
| Seeds & nuts |  | 0.25 |  |
| Sauces and dressings | 0.41 |  |  |
| Fried potatoes | 0.48 | -0.25 |  |
| Vegetables |  | -0.43 |  |
| Vegetable dishes |  | 0.20 | -0.24 |
| Snacks |  |  | -0.36 |
| Cakes & Sweets |  |  |  |
| Alcoholic beverages | 0.45 |  |  |
| Vegemite |  |  | -0.24 |
|  |  |  |  |
| **Variance Explained (%)** | **7.2** | **5.7** | **5.3** |

Absolute values <0.2 are not shown
